# An Approximate Method for Exploring Nonradiative Decay Pathways From Highly Excited States of Lanthanide Complexes: Application to Luminescent Cerium Complexes

**DOI:** 10.1002/jcc.70327

**Published:** 2026-02-07

**Authors:** Soshi Ikuta, Taichi Inagaki, Miho Hatanaka

**Affiliations:** ^1^ Graduate School of Science and Technology Keio University Yokohama Kanagawa Japan; ^2^ Institute for Molecular Science Okazaki Aichi Japan

**Keywords:** 4f–5d transition, charge‐transfer excitation, density functional theory (DFT)

## Abstract

The exploration of minimum energy crossing points (MEXs) between potential energy surfaces (PESs) is essential for understanding nonradiative decay mechanisms and plays a key role in the design of photofunctional materials. In lanthanide (Ln^3+^) complexes, however, the presence of open‐shell 4f^*N*^ electrons leads to quasi‐degenerate electronic states, making MEX searches particularly challenging. To describe the PESs of 4f‐5d or charge‐transfer excited states (i.e., 4f^*N*−1^X excited states) of Ln^3+^ complexes, we propose a new approximation, the ion energy shift (IES) method. In this approach, the 4f^*N*−1^X excited state is represented using density functional theory (DFT) with the large‐core relativistic effective core potential (RECP) for Ln^4+^, which has a higher formal charge than the actual ion (Ln^3+^), and the PES is shifted to reproduce the target excitation energy. In this study, we validate the IES method against the multiconfigurational wavefunction results and apply it to elucidate the origin of the different excited‐state lifetimes of hydrated Ce^3+^ complexes with and without coordination of a carboxylate ligand.

## Graphical abstract

We present the ion energy shift method, a new approach that enables the optimization of critical structures on and between the ground state and the 4f^*N*−1^X excited state, involving either 4f–5d or charge‐transfer excited states in lanthanide complexes.

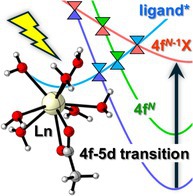

## 1 Introduction

For the rational design of photofunctional materials, it is essential to control excited‐state lifetimes, as well as excitation energies and oscillator strengths. One of the key factors determining excited‐state lifetime is the nonradiative deactivation from the excited state, which is mainly governed by the stability of minimum energy crossing point (MEX) between the potential energy surfaces (PESs) of the ground and excited states, namely conical intersection or minimum‐energy seam of crossing [1, 2, 3]. Accordingly, quantum chemical calculations of MEXs have been extensively performed for a wide range of molecular systems. Since conical intersections cannot be described using conventional time‐dependent density functional theory (TDDFT), advanced approaches such as multiconfigurational wavefunction methods [4, 5] or spin‐flip TDDFT method [6] have been employed. However, even these state‐of‐the‐art methods have proven inadequate for the calculation of MEXs in lanthanide (Ln) complexes. The challenge arises from the open‐shell 4f electrons of Ln, which are not affected by surrounding environments due to the shielding by the outer closed‐shell 5s and 5p orbitals. As a result, the ligand field splitting of the 4f^*N*^ states is small, and for a ground state labeled by a ^2*S*+1^ *L*_*J*_ term, a (2*J* + 1)‐fold pseudo‐degeneracy is retained. The excited states also exhibit a similar pseudo‐degeneracy. Due to this, even multiconfigurational wavefunction methods face substantial difficulties in exploring the PESs and locating MEXs.

To address this issue, the energy shift (ES) method has been proposed as a practical alternative [7]. This method enables theoretical investigation of nonradiative decay processes from 4f–4f excited states through ligand‐localized excited states by approximately representing the PES of 4f–4f excited states using the PES of the ground state with the ES parameter. Originally developed for exploring MEXs between 4f–4f excited states and other states in Ln complexes, the ES method has also been adapted to organic molecules to explore the vicinity of conical intersections between the singlet ground and excited states (S_0_ and S_1_) using the DFT and TDDFT frameworks [8, 9, 10, 11].

However, the ES method is not applicable to the emerging class of Ln photofunctional complexes that utilize 4f–5d excited states and charge‐transfer (CT) excited states. A representative example is the Ce^3+^ complex. Since the early 2010s, Ce^3+^ complexes have attracted growing attention as photo‐redox catalysts [12, 13, 14]. In these systems, photoexcitation induces a 4f–5d transition, and the resulting excited‐state energy is utilized to promote redox reactions. To evaluate the photostability and reactivity of such systems, it is essential to understand the deactivation pathways from 4f–5d excited states. The 4f–5d excited states of Ce^3+^ systems are also expected to be applied to organic light‐emitting diodes (OLEDs) [15, 16] and optical materials using delayed fluorescence [17]. Yet, theoretical studies of these excited states remain scarce due to the complexity of their electronic structures. As with other Ln systems, the 4f orbitals in Ce^3+^ complexes exhibit pseudo‐degeneracy, leading to intricate crossings with 5d‐excited states. Furthermore, the ES method, which incorporates the 4f electrons into the effective core potential (ECP), fails to capture the nature of 4f–5d and CT transitions. These limitations highlight the pressing need for a new computational framework capable of describing the PESs of 4f–5d and CT excited states.

In this study, we propose a new approximate approach—the ion energy shift (IES) method—for describing the PES of 4f^*N*−1^X state, including 4f–5d and CT excited states. To validate the accuracy of the IES method, we perform benchmark calculations on the CeOH^2+^ model complex and compare the results obtained by the IES method and the multistate formulation of second‐order multireference perturbation theory (MS‐CASPT2) [18, 19]. Finally, we demonstrate the applicability of the IES method by analyzing the difference in excited‐state lifetimes between Ce^3+^ hydrate and carboxylate complexes [20].

## 2 Theory

### 2.1 ES Method

**FIGURE 1:**
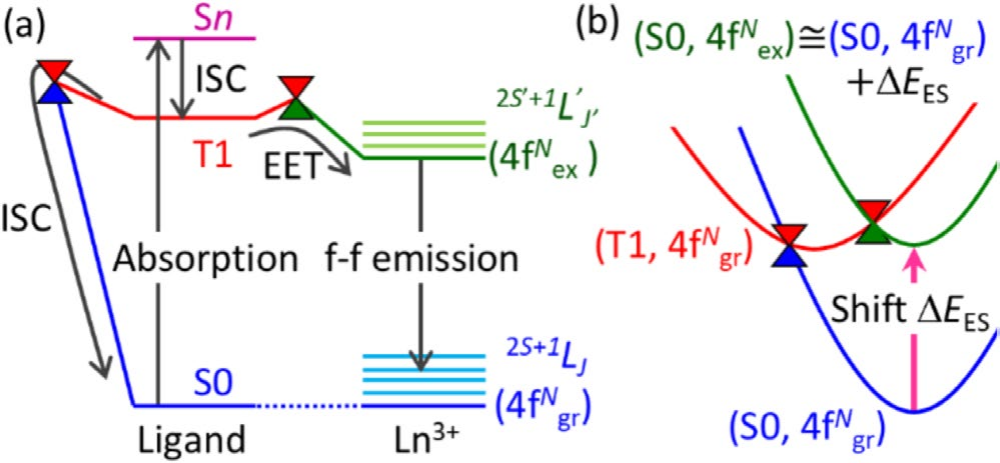
(a) The Jablonski diagram of Ln^3+^ with a photo‐antenna ligand and (b) the schematic illustration of the ES method [7]. ^2*S*+1^ *L*_*J*_ (4f^*N*^_gr_) and ^2*S*′+1^ *L'*_*J′*_ (4f^*N*^_ex_) correspond to the ground and the 4f–4f excited states of Ln^3+^ moiety, respectively. The PESs of the ground state, ligand‐localized T1 state, and the 4f–4f excited state are in blue, red, and green, respectively. The ES parameter Δ*E*_ES_ is the excitation energy of the 4f–4f excited state.

First, we describe how the ES method [7] enables the computation of nonradiative decay pathways from the 4f–4f excited state of Ln^3+^ complexes. The mechanism of 4f–4f luminescence of Ln^3+^ complexes has traditionally been explained based on the Jablonski diagram shown in Figure 1a [21, 22, 23, 24, 25]. First, one of the ligands, called the photo‐antenna ligand, absorbs the light and populates a ligand‐localized triplet (T1) state. If the energy level of this T1 state is higher than that of the 4f–4f excited state, excitation energy transfer (EET) from the ligand to Ln^3+^ occurs, followed by luminescence via the 4f–4f transition. Since the lifetime of the parity‐forbidden 4f–4f excited state is relatively long, nonradiative decay via backward EET and intersystem crossing (ISC) from the T1 state to the ground state may occur. The rates of these processes are determined by the stabilities of the corresponding MEXs. However, as mentioned above, locating these MEXs is computationally demanding.

To address this, we focus on the shape of PESs. Because 4f electrons are well shielded from the surroundings by the outer closed‐shell 5s and 5p electrons, the PES of the 4f–4f excited state has a shape nearly identical to that of the ground state. Due to the same reason, the 4f–4f excitation energy is independent of the surroundings. Therefore, the PES of the 4f–4f excited state can be approximated by vertically shifting the ground‐state PES by an amount equal to the experimental excitation energy, which is called the ES parameter (see Figure 1b). In this framework, the electronic configuration of Ln^3+^ center remains the same (^2*S*+1^ *L*_*J*_) for all three key states: the ground state, the ligand‐localized T1 state, and the 4f–4f excited state. This allows us to avoid explicitly treating the 4f electrons when computing energy differences among these states. As a result, we can employ the large‐core Stuttgart–Dresden relativistic effective core potential (RECP) [26, 27] for the Ln^3+^ center, in which [Kr]4d^10^4f^*N*^ electrons were included in the RECP. Using this large‐core RECP, the three relevant states can be computed as follows: the ground state as the lowest singlet state, the ligand‐localized T1 state as the lowest triplet state, and the 4f–4f excited state as the lowest singlet shifted by the ES parameter. All these states can be calculated using conventional ground‐state calculation methods, such as the DFT method. Consequently, the MEXs relevant to the EET from the ligand‐T1 and 4f–4f excited states, as well as the ISC from the ligand‐T1 to the ground state, can be efficiently explored at the DFT level.

The ES method has been applied to elucidate the origin of changes in the 4f–4f emission intensities of Ln^3+^ complexes [7, 28, 29, 30, 31] and polymers [32], in different environments, such as with different ligands or at different temperatures. Furthermore, the ES method successfully provided ligand design strategies to achieve the desired luminescence properties [28, 32]. However, this method is not applicable to systems involving excited states with different numbers of 4f electrons, such as 4f–5d excited states or CT excited states between Ln^3+^ and ligands.

### 2.2 IES Method

**FIGURE 2:**
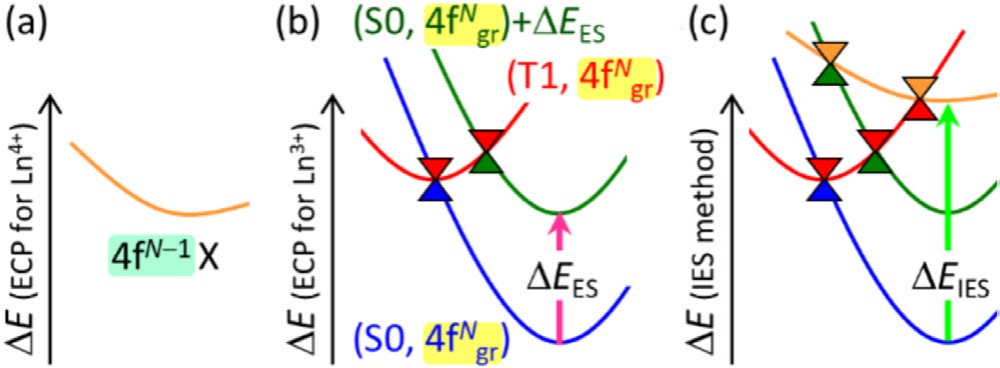
Schematic illustration of the IES method. (a) PES of the 4f^*N*−1^X excited state, in which 4f^*N*−1^ electrons (highlighted in light green) are included in the large‐core RECP for Ln^4+^, is shown in orange. (b) PESs of the ground state, the ligand‐localized T1 state, and the 4f–4f excited states shown in blue, red, and green, respectively, are described with the ES method. 4f^*N*^ electrons (highlighted in yellow) are included in the large‐core RECP for Ln^3+^. Δ*E*_ES_ is the ES parameter. (c) PESs of the four states shown in (a) and (b), where the energy level of the 4f^*N*−1^X excited state is shifted to reproduce its excitation energy (Δ*E*_IES_).

To overcome the limitations of the ES method, we propose using the large‐core RECP of Ln^4+^, which has a larger formal charge than the actual Ln^3+^, to describe the shapes of the 4f^*N*−1^X excited state in Ln^3+^ complexes, such as the 4f–5d excited state or metal‐to‐ligand charge transfer (MLCT) excited state. By employing the RECP for Ln^4+^, which treats [Kr]4d^10^4f^*N*−1^ as the core [33], in the calculation of the Ln^3+^ complexes, the remaining electron that originally occupied a 4f orbital in the ground state is promoted to another valence orbital. As a result, the valence electronic configuration of this state matches that of the target 4f^*N*−1^X excited state. This allows us to approximate the shape of the lowest 4f^*N*−1^X excited state using the lowest doublet state computed with the large‐core RECP for Ln^4+^ (see Figure 2a).

In this case, however, the absolute energies cannot be directly compared to those obtained using the ES method, which employs the large‐core RECP for Ln^3+^ (see Figure 2b). Therefore, we introduce an ES to the PES of the 4f^*N*−1^X excited state to reproduce the corresponding excitation energy at the global minimum (GM), which can be taken from experimental measurements or high‐level theoretical calculations (see Figure 2c). The concept of this approach is based on the ES method but involves using the RECP of a different ion; hence, we refer to it as the IES method. Similarly, the RECP of Ln^2+^ [26, 27] can be used to describe ligand‐to‐metal charge transfer (LMCT) excited states. Note that it is not obvious which geometry should be selected to obtain excitation energy for the ES parameter. In this study, we selected the excitation energy at the GM because we were interested in MEXs near the Frank–Condon region.

## 3 Computational Details

All PESs described using the ES and IES methods were calculated by DFT with the ωB97XD functional [34]. The cc‐pVDZ basis set [35] was used for H, C, and O atoms throughout. The basis set and RECP used for Ce depended on the electronic state. For the ground and ligand‐localized T1 states, the large‐core RECP for Ce^3+^ (ECP47MWB) and the corresponding (7s6p5d)/[5s4p3d] basis set [26, 27] were used. By using this RECP, the ground and T1 states were computed as singlet and triplet, respectively. For the 4f^0^X excited state, the large‐core RECP for Ce^4+^ (ECP46MWB) and the corresponding (7s6p5d)/[4s3p3d] basis set [33] were used. With this RECP, the 4f^0^X excited state was calculated as doublet. The ES parameter for the IES method (IES parameter) was determined to reproduce the excitation energy at the optimized geometry on the ground state.

For CeOH^2+^, the IES parameter was calculated at the MS‐CASPT2 level. The molecular orbitals used for MS‐CASPT2 were optimized at the eight state‐average (SA) complete active space self‐consistent field (CASSCF) method [36, 37] with the active space of (1e,17o). (See the details of the 17 active orbitals in Figure S1.) The electronic state of Ce^3+^ with 4f^1^ configuration (^2^ *F*) includes seven states, thus all the 4f^1^ and one 4f^0^X state, resulting in eight states, were considered in the SA‐CASSCF calculation. The characters of these eight states were confirmed by the singly occupied natural orbitals (SONOs) shown in Figure S2. The small‐core RECP and its corresponding basis set (ECP28MWB_ ANO) [38, 39] were used for Ce. The cc‐pVDZ basis set was used for other atoms. To validate the IES method, the critical structures of CeOH^2+^ were optimized at the same level of MS‐CASPT2 calculation.

For Ce^3+^ hydrate and carboxylate, the IES parameters were calculated at TDDFT with the ωB97XD functional. The basis set and RECP used for the TDDFT were the same for the MS‐CASPT2 above (i.e., ECP28MWB_ANO and cc‐pVDZ). The solvation effect was included by the polarized continuum model (PCM) with a dielectric constant of 78.3553 (water) [40] both for the TDDFT and DFT with the ES and IES methods.

The geometry optimization of local minima (LMs), transition states (TSs), and MEXs on and between the ground and excited states, and the intrinsic reaction coordinate (IRC) calculation [41], used to confirm the TSs, were performed via the global reaction route mapping (GRRM) program [42]. The energies and energy derivatives at the DFT and TDDFT used in the GRRM program were computed with the Gaussian16 program [43]. Those at the MS‐CASPT2 were computed with the Molpro program [44, 45, 46].

## 4 Results and Discussion

### 4.1 Validation of the IES Method

**FIGURE 3:**
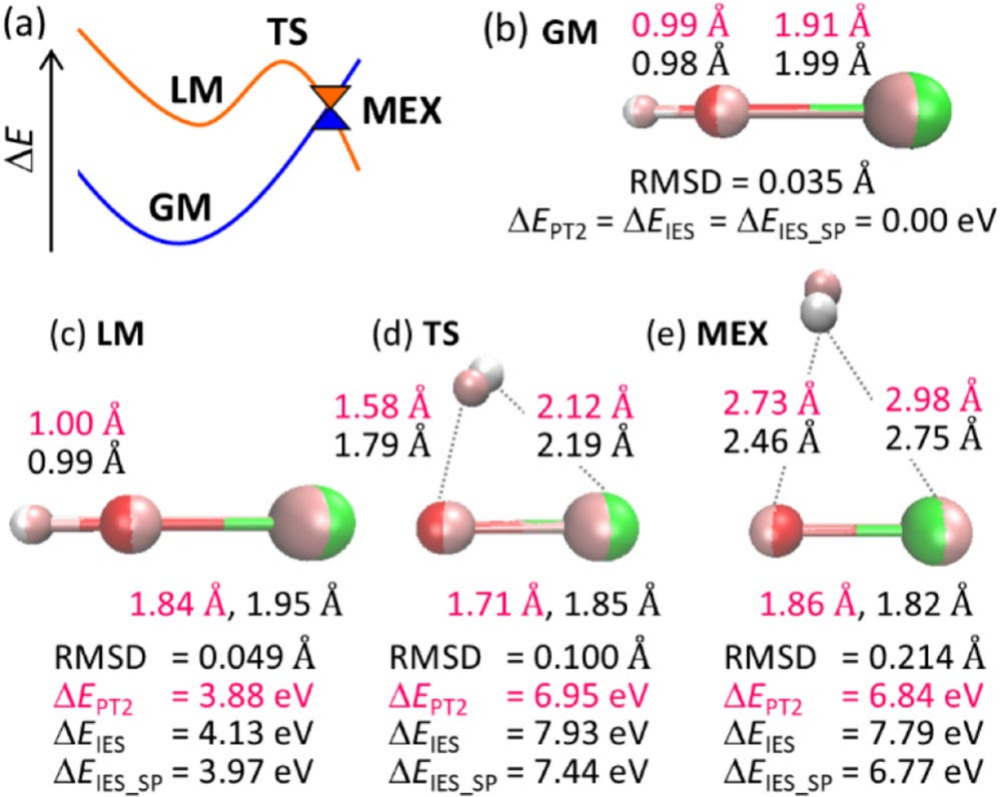
Overview of the PESs of the ground state (in blue) and the 4f^0^X excited state (in orange) of CeOH^2+^ (a). The geometries of GM (b), LM (c), TS (d), and MEX (e), optimized using the MS‐CASPT2 and the IES methods, are shown in pink and in atomic‐color‐code (Ce: light green, O: red, H: white), respectively. The atomic distances (in Å) obtained with the MS‐CASPT2 and IES methods are in pink and black, respectively. The RMSDs between the MS‐CASPT2‐ and IES‐optimized geometries are given in Å. Δ*E*_PT2_, Δ*E*_IES_, Δ*E*_IES_SP_ represent the relative energies (in eV) obtained with the MS‐CASPT2, IES method, and single‐point MS‐CASPT2 at the IES‐optimized geometry, respectively. All relative energies are referenced to the GM energy. In the MS‐CASPT2 calculation, the MEX between the highest 4f^1^ and the lowest 4f^0^X (i.e., the sixth and seventh excited states) was located, whilst in the IES method, the MEX between the ground state and the lowest 4f^0^X was obtained. (See the details in Figure S4.)

The IES method was validated by comparing the geometries of CeOH^2+^ optimized at the MS‐CASPT2 level. Specifically, we optimized the four critical structures shown in Figure 3a: GM on the ground state, LM on the 4f^0^X excited state, MEX between the 4f^1^ and 4f^0^X states, and TS on the 4f^0^X state, which was found between LM and MEX, using both the IES and MS‐CASPT2 methods. (See the PESs of the 4f^1^ and 4f^0^X states in Figure S3 and Table S1.) Both methods yielded linear geometries for the GM and LM, and triangular geometries for the TS and MEX, in which the H atom was partially dissociated from the O atom (see Figure 3b–e). The structural differences between the two methods were evaluated using the root‐mean‐square deviation of atomic positions (RMSD). The RMSD values for the GM and LM were as small as 0.035 Å and 0.049 Å, respectively, whereas those for the TS and MEX were larger but still remained below 0.214 Å. These results indicate that the structures farther from the one used to determine the IES parameter exhibit greater discrepancies from the true structures. Furthermore, to examine the effect of the IES parameter on the optimized geometry, the MEX was reoptimized using the updated IES parameter that reproduces the excitation energy at the MEX geometry. This adjustment reduced the RMSD only slightly (0.214 Å → 0.207 Å), indicating that, for qualitative geometry optimization, it is sufficient to set the IES parameter so as to reproduce the excitation energy at the GM.

Next, we compared the relative energies at the optimized structures obtained with the IES and MS‐CASPT2 methods. The difference in relative energies between the two methods was 0.25 eV, 0.98 eV, and 0.95 eV for the LM, TS, and MEX, respectively (see Δ*E*_PT2_ and Δ*E*_IES_ in Figure 3b–e), indicating that relative energies tend to be increasingly overestimated as the structure deviated farther from the GM. This overestimation could be attributed to the neglect of electron correlation between the 4f and valence electrons, which becomes more significant near the MEX. To mitigate this effect, single‐point MS‐CASPT2 calculations were performed at the IES‐optimized geometries. As a result, the energy deviations were reduced to 0.09 eV, 0.49 eV, and −0.07 eV for the LM, TS, and MEX, respectively (see Δ*E*_IES_SP_ in Figure 3b–e). This trend was not unique to MS‐CASPT2 but was also observed in other methods, such as the TDDFT method, as shown in Figure S5. From the perspective of computational cost, TDDFT is a more practical choice than MS‐CASPT2 for energy evaluation. Thus, a practical approach to exploring nonradiative pathways from the 4f^*N*−1^X excited state is the geometry optimizations using the IES method with an IES parameter determined at the GM geometry using TDDFT, followed by single‐point calculations at the same level.

**FIGURE 4:**
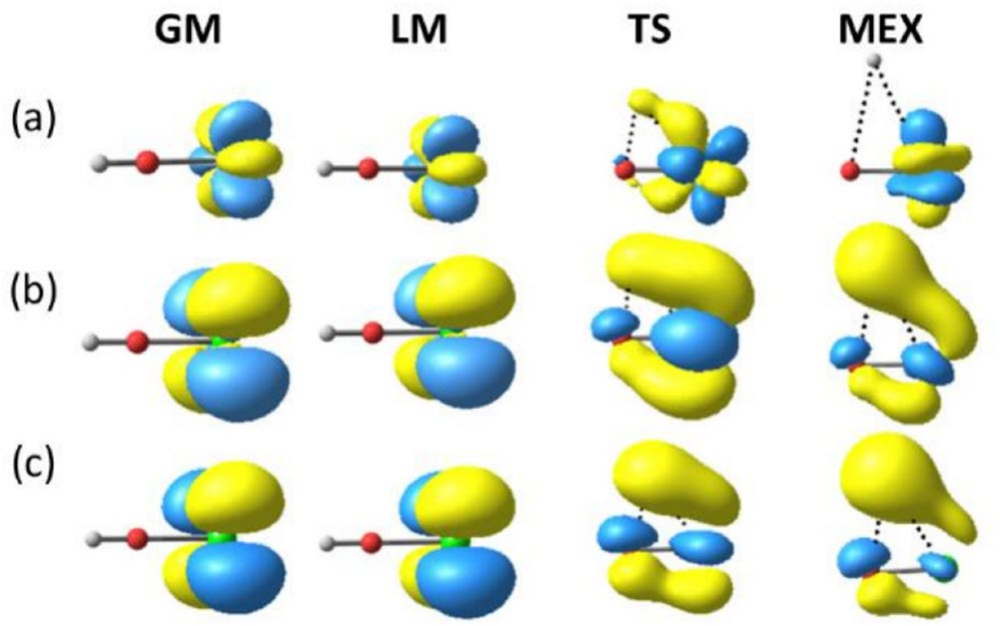
SONOs of the ground state (a) and the 4f^0^X excited state (b) at the MS‐CASPT2 level and SOMOs of the excited state at the IES level (c) for the four critical structures shown in Figure 3. Ce, O, and H are in light green, red, and white, respectively.

From another perspective, it is important to examine whether the IES method captures the nature of the electronic states. To analyze the nature of the electronic states at the optimized geometries, we examined the SONOs and molecular orbitals (SOMOs) obtained at the MS‐CASPT2 and IES levels, respectively. As shown in Figure 4a, all SONOs of the ground state were attributed to 4f orbitals on Ce, supporting the premise of the IES method that treats the 4f electron within the RECP. In contrast, the SONOs of the excited state varied depending on the structure. As shown in Figure 4b, the SONOs for the GM and LM were localized 5d orbitals on Ce, whereas significant mixing of the H 1s and O 3p orbitals with the Ce 5d orbital was observed for the TS and MEX. In other words, the nature of the excited state evolved from a 4f–5d excitation to an MLCT excitation along the pathway from the GM to MEX. This change in excited‐state character was consistently reproduced by the SOMOs calculated at the IES level (i.e., DFT with large‐core Ce^4+^ RECP), as shown in Figure 4c. The ability of DFT with the large‐core RECP to capture trends in electronic structure likely underlines the good agreement observed between the IES and MS‐CASPT2 methods.

It should be noted that the effect of spin‐orbit (SO) interaction, which is not considered in this study, is expected to be small. The SO splittings of Ce^3+^ are 0.28 eV for the 4f^1^ states and 0.31 eV for the 5d^1^ states [47], which are comparable to the ligand field splitting of 4f^1^ calculated at the MS‐CASPT2 level (see the PESs of 4f^1^ states in Figure S3 and Table S1). Considering that MEX with the 4f^0^X state is very similar regardless of which 4f^1^ state is considered (see Figure S4), the influence of SO splitting on both the MEX geometry and its energy is expected to be minor. From the viewpoint of the mixing of different spin states in the wavefunction, the effect of SO interaction on the shapes of PESs is also expected to be small. In general, the PESs of Ln 4f^*N*^ states are scarcely affected by whether SO interaction is included, because the PESs of other 4f^*N*^ states with different spin multiplicities that are mixed in through SO interaction have nearly identical shapes. In the case of Ce^3+^ systems, the target states, such as 4f^1^ and 5d^1^, are doublet, and the states with different spin multiplicities, such as quartet states, arise from 5p →4f excitations (5p^5^4f^2^) or ligand‐to‐4f CT excitations. Since these states lie much higher in energy than the target doublet states (4f^1^ or 4f^0^X), their mixing into the target states is expected to be negligible. Consequently, the influence of SO interaction on the shapes of the PESs of the target doublet states is also expected to be minimal.

### 4.2 Application of the IES Method to Real Systems

Next, the IES method was applied to study the nonradiative decay process of real Ce^3+^ complexes. It is well known that hydrated Ce^3+^ exhibits strong luminescence originating from the 4f–5d transition; however, this luminescence is quenched upon complexation with carboxylates [48, 49]. Time‐resolved studies of the excited‐state decay of Ce^3+^ in the carboxylate complex revealed a fast component with a lifetime of 1–2 ns, in addition to a slow component with a lifetime of 40 ns, which was attributed to the hydrated Ce^3+^ [20]. To clarify the origin of the fast nonradiative decay of the Ce carboxylate complex, we computed the potential energy profiles of [Ce(H_2_O)_7_(OAc)]^2+^ and [Ce(H_2_O)_8_]^3+^ using the IES method with the IES parameter obtained at TDDFT level.

**FIGURE 5:**
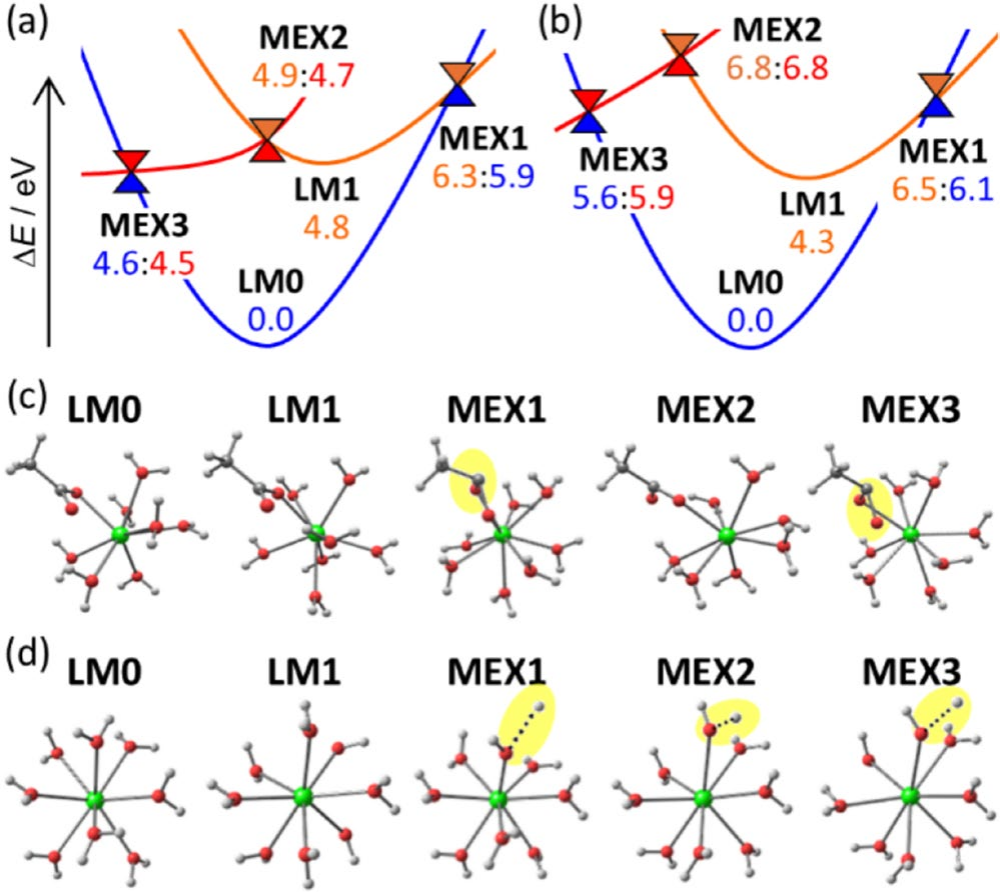
Potential energy profiles Δ*E* (in eV) of [Ce(H_2_O)_7_(OAc)]^2+^ (a) and [Ce(H_2_O)_8_]^3+^ (b). Blue, red, and orange represent the ground state, ligand‐localized T1 state, and 4f–5d excited state, respectively. The relative energies in blue, red, and orange were obtained by the single‐point calculations of the DFT in doublet, the DFT in quartet, and the TDDFT in doublet, respectively, with the small‐core RECP at the IES‐optimized geometries, thus two energies at the MEXs were slightly different. Geometries at the LMs and MEXs of [Ce(H_2_O)_7_(OAc)]^2+^ (c) and [Ce(H_2_O)_8_]^3+^ (d). The representative structural changes were highlighted in yellow. The spin density at each geometry is shown in Figure S6.

As shown in Figure 5a,b, LMs and MEXs were located on and between the ground state, 4f^0^X excited state, and ligand‐localized T1 state. The LM on the 4f^0^X state (LM1) had a similar geometry to the LM on the ground state (LM0) for both complexes as shown in Figure 5c,d. The relative energies of the LM1 were 4.8 eV for the carboxylate complex (see Figure 5a) and 4.3 eV for the hydrate complex (see Figure 5b). These similar values were attributed to the 4f–5d excitation character of this state (see Figure S6), which is only weakly influenced by the ligands. The geometry of the MEX between the 4f^0^X and ground states (MEX1) was found for both complexes. The MEX1 exhibited a pyramidal structure for the carboxylate and a stretched OH bond for the hydrate (see the yellow highlights in Figure 5c,d). Though the structural changes (i.e., reaction coordinates) differed for the two complexes, both energies exceeded 6.0 eV, which were too high to be accessible from LM1.

In contrast, the character of the ligand‐localized T1 state depended strongly on the complex: the excitation was localized on one of the water molecules in the hydrate complex and on the carboxylate ligand in the carboxylate complex (see Figure S6). This difference led to markedly different stability of the MEX2, which was the MEX between the T1 and 4f^0^X states. For the carboxylate complex, the energy difference between MEX2 and LM1 was only 0.1 eV, whereas that for the hydrate complex was as large as 2.5 eV. Thus, the EET from the Ce 5d orbital to ligand could occur only in the carboxylate complex. Once the system reached the T1 state, ISC from the T1 to the ground state through MEX3 could proceed without a barrier. Therefore, the shorter excited‐state lifetime of the carboxylate complex can be attributed to the low‐lying ligand‐localized T1 state. From a structural perspective, the geometry of the carboxylate moiety changed from planar to pyramidal along the pathway from LM1 to MEX3. Thus, this result indicates that out‐of‐plane bending of the carboxylate moiety induced the quenching of the 4f–5d emission.

## 5 Conclusions

In this study, we proposed a new approximate approach, termed the IES method, for exploring nonradiative decay processes from the 4f^*N*−1^X excited state of Ln complexes, including both 4f–5d and MLCT excited states. This method enabled the optimization of MEXs between the ground and 4f^*N*−1^ excited states, as well as other critical structures such as LMs and TSs along the pathway from the Franc‐Condon region to the MEX. The essence of the IES method lies in describing the PES of the 4f^*N*−1^X excited state without explicitly treating the 4f^*N*−1^ electrons, by placing them in the large‐core RECP of a higher formal charge than the actual ion (e.g., Ln^4+^ RECP for an Ln^3+^ complex). With this RECP, the numbers of 4f and X electrons are constrained to *N* − 1 and 1, respectively, enabling the PES of the 4f^*N*‐1^X excited state to be described by DFT. In addition, the PESs of the ground state and the ligand‐localized triplet state, both of which contain 4f^*N*^ electrons, were described as the lowest singlet and lowest triplet states, respectively, using DFT with the large‐core RECP that places all 4f^*N*^ electrons in the RECP. Since energies calculated using different RECPs cannot be directly compared, the PES of the 4f^*N*−1^X excited state was shifted to reproduce the 4f^*N*−1^X excitation energy for a given structure.

The validity of the IES method was confirmed by comparing with MS‐CASPT2 and TDDFT calculations. Although the structural difference between the IES method and the conventional methods (MS‐CASPT2 and TDDFT) increased with structural deviation from the GM, the structural error in the CeOH^2+^ model complex remained within an RMSD of 0.214 Å. Moreover, the energetics could be refined by performing single‐point MS‐CASPT2 or TDDFT calculations at the IES‐optimized geometries.

Because the IES method is based solely on DFT, it is applicable to large Ln systems. As a demonstration, we applied it to [Ce(H_2_O)_8_]^3+^ and [Ce(H_2_O)_7_(OAc)]^2+^. The former exhibits strong luminescence originating from the 4f–5d transition, whereas the latter shows a short lifetime for the 4f–5d excited state. The IES method revealed that in the Ce^3+^ hydrate, the energies of the MEXs between the 4f–5d excited state and other states were very high, making nonradiative decay unlikely. In contrast, in the Ce carboxylate, the T1 state localized on the carboxylate moiety lay at much lower energy and intersected with the 4f–5d excited state, thereby facilitating nonradiative decay.

The present study demonstrates that the IES method can qualitatively describe nonradiative decay pathways in Ce complexes at a computational cost comparable to conventional DFT. This approach is therefore expected to be applicable to a wide range of Ln systems, including larger molecular complexes and extended coordination networks relevant to photofunctional materials and photocatalysis. In particular, the ability to locate MEXs and critical geometries provides a useful tool for guiding the design of luminescent and photocatalytic Ln complexes. Nevertheless, several limitations remain. The accuracy of the IES method depends on the quality of the IES parameter, and the neglect of explicit electron correlation between 4f and valence electrons may lead to quantitative errors. Future work should therefore focus on systematically assessing these limitations and extending the approach to other classes of Ln complexes.

## Author Contributions

The manuscript was written through the contributions of all authors.

## Funding

This work was supported by Japan Society for the Promotion of Science, JP24H01094 and Japan Science and Technology Agency, JPMJPF2221.

## Conflicts of Interest

The authors declare no conflicts of interest.

## Supporting information


**Data S1:** jcc70327‐sup‐0001‐Supinfo.pdf.

## Data Availability

The data that supports the findings of this study are available in the Supporting Information of this article.
