# Supplementary material for: An Approximate Method for Exploring Nonradiative Decay Pathways From Highly Excited States of Lanthanide Complexes: Application to Luminescent Cerium Complexes
Source: J Comput Chem. 2026 Feb 7;47(5):e70327. doi: 10.1002/jcc.70327 (PMC12882562; doi:10.1002/jcc.70327)
Supplement: Supplementary file 1 — Data S1: jcc70327‐sup‐0001‐Supinfo.pdf. [file JCC-47-0-s001.pdf]

Supporting Information for

An approximate method for exploring nonradiative decay pathways from highly excited states of lanthanide complexes: application to luminescent cerium complexes

Soshi Ikuta,[†] Taichi Inagaki,[†] and Miho Hatanaka[†, ‡,*]

[†]Graduate School of Science and Technology, Keio University. 3-14-1 Hiyoshi, Kohoku-ku, Yokohama, Kanagawa, 223-8522, Japan.

[‡]Institute for Molecular Science.38 NishigoNaka, Myodaiji, Okazaki, Aichi, 444-8585, Japan.

*E-mail: miho_hatanaka@keio.jp

Contents

1. Details of calculated results S2
2. Cartesian coordinates and energies of critical structures S7

1. Details of calculated results

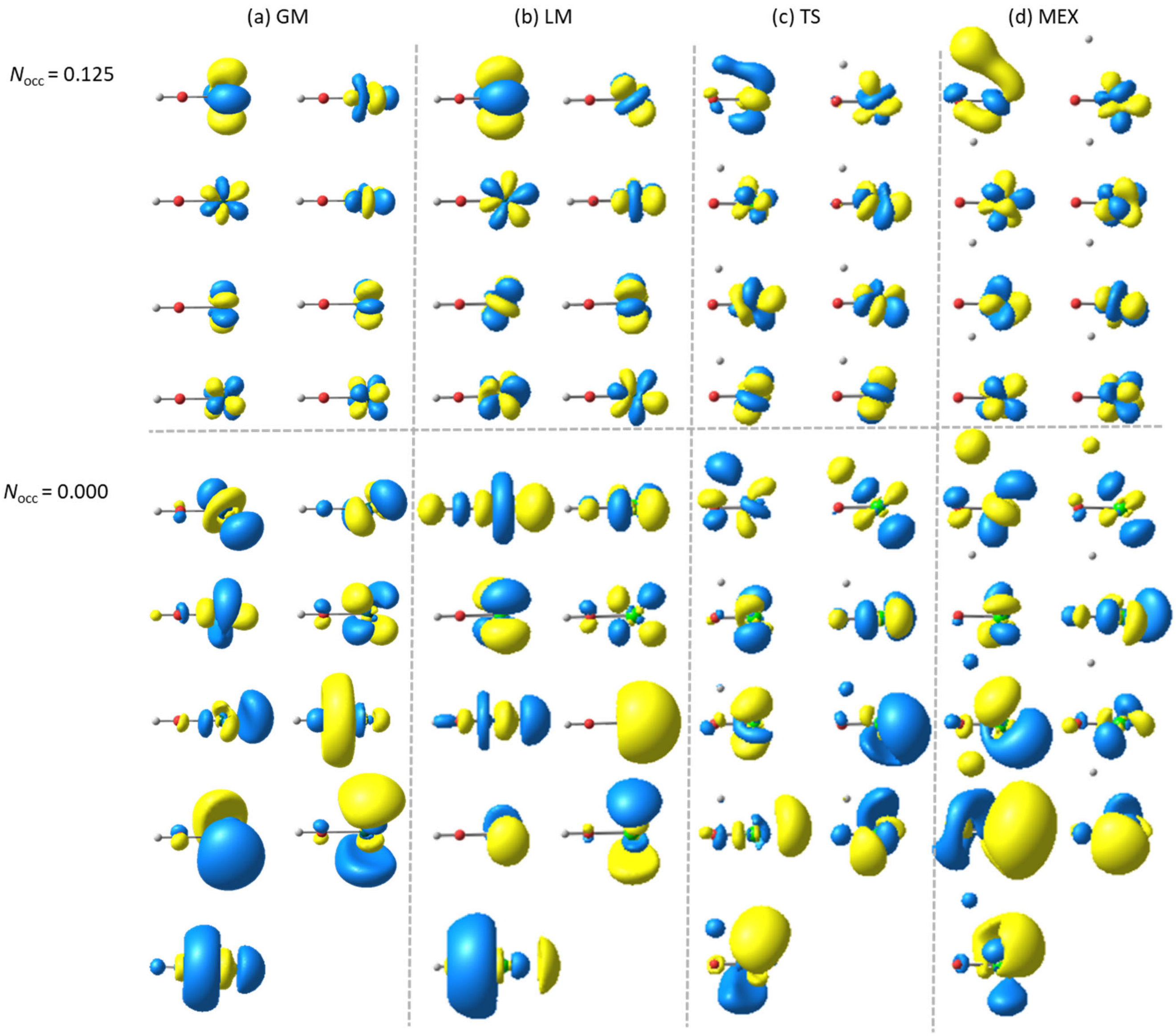

Figure S1. The active orbitals used in the 8SA-CASSCF(1e,17o) calculations for the GM (a), LM (b), TS (c) and MEX (d) geometries optimized at the MS-CASPT2 level. N_{occ} represents the occupation number calculated for the eight-averaged-state. Although at least seven Ce 4f orbitals and five Ce 5d orbitals were initially considered necessary for inclusion in the active space, only eight orbitals exhibited non-zero occupation numbers. These eight active orbitals consist of seven Ce-localized 4f orbitals and one 5d orbital, the latter showing mixing with the H 1s orbital at the TS and MEX geometries. Nearly identical active orbitals were also obtained for the four geometries optimized at the IES level.

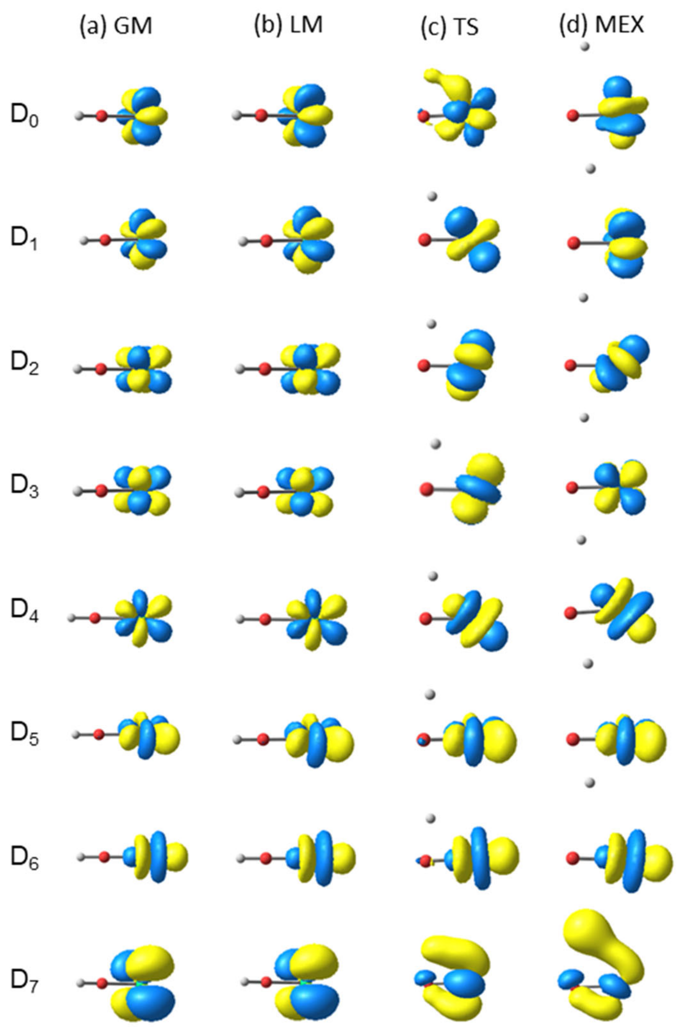

Figure S2. The singly occupied natural orbitals (SONOs) of the eight doublet states, from the ground state D_0 to the seventh excited state D_7, calculated at the four critical geometries—GM (a), LM (b), TS (c), and MEX (d)—optimized at the MS-CASPT2 level. For all four geometries, the SONOs of the seven lower-lying states D_n ($n = 0, 1, \cdots, 6$) are Ce-localized 4f orbitals, indicating predominant $4f^1$ electronic configurations. In contrast, the SONO of D_7 corresponds to a Ce-localized 5d orbital at the GM and LM geometries, while the ligand contribution increases at the TS and MEX geometries. Accordingly, the D_7 state is characterized as a $4f^0X$ configuration: specifically, $5d^1$ at GM and LM, and $5d^1$ with partial metal-to-ligand charge transfer (MLCT) at TS and MEX. A nearly identical trend was also observed for the eight states calculated at the four geometries optimized at the IES level.

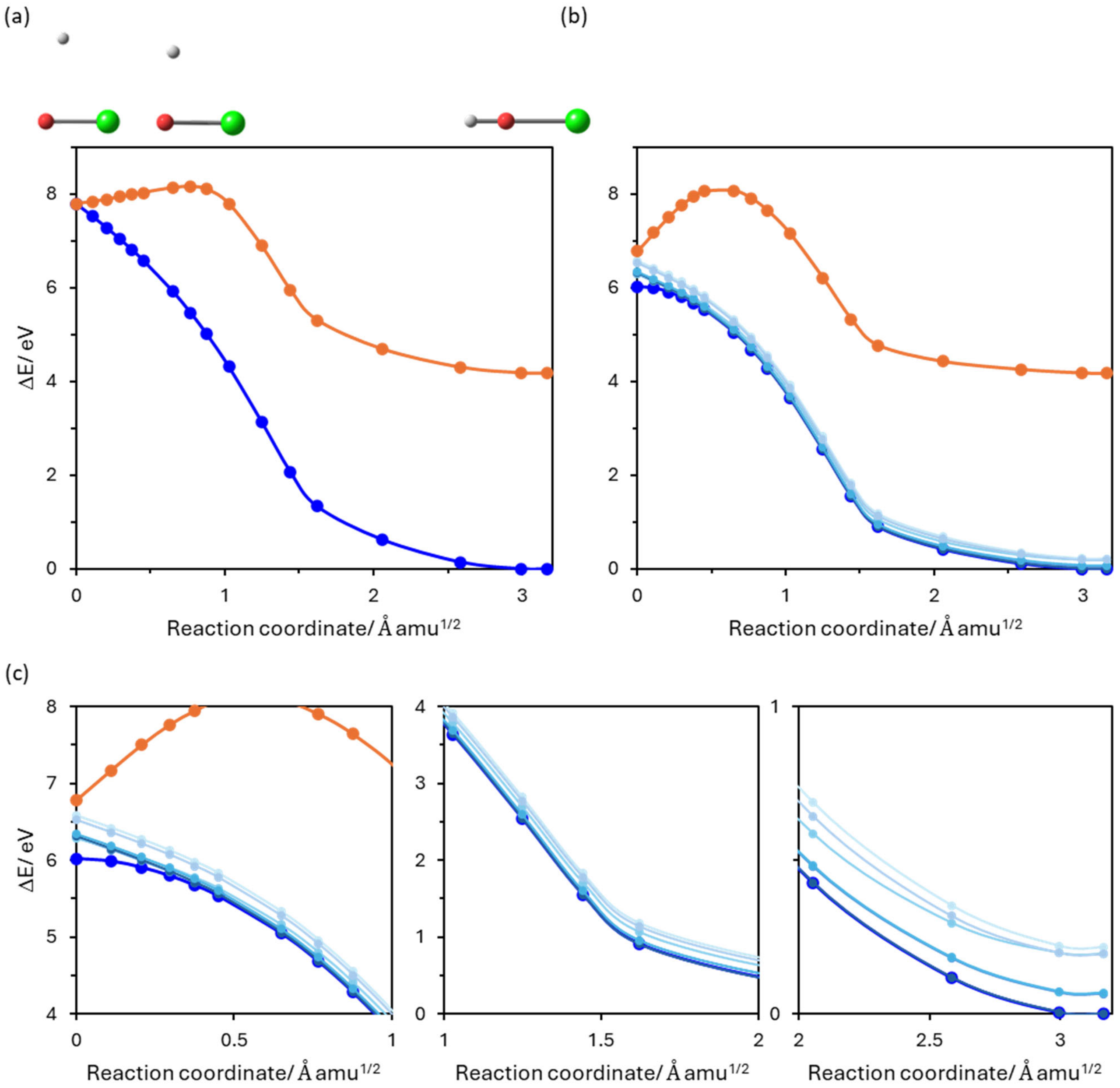

Figure S3. The potential energy curves (PECs) along the reaction coordinate from the IES-optimized MEX to the GM obtained through the IRC calculation on the ground state. The relative energies Δ*E* (in eV) of the ground state (in blue) and the $4f^0X$ excited state (in orange) were calculated at the IES level of theory (a). The PECs with the same reaction coordinate and the Δ*E* (in eV) of the seven $4f^1$ states (in blue and light blue) and a $4f^0X$ excited state (in orange) obtained by the single-point MS-CASPT2(1e,17o) calculations (b). An enlarged views of (b) are also shown (c). As shown in (b) and (c), the energy splitting among the $4f^1$ states is much smaller than the Δ*E* of $4f^0X$ at the GM geometry, indicating that the seven $4f^1$ states can be considered as quasi-degenerate. The energy splitting among the $4f^1$ states is 0.22 eV at the GM geometry, gradually increases to 0.30 eV near the MEX (reaction coordinate = 0.38 Å $amu^{1/2}$), and then increases sharply to 0.55 eV at the MEX. This behavior indicates that the seven $4f^1$ states are approximately parallel along the reaction coordinate, although this approximation is slightly violated in the vicinity of the MEX. (See the detailed values in Table S1.)

Table S1. The relative energies ΔE (in eV) of the eight doublet states (D_n; n = 0,⋯, 7) and the energy splitting (in eV) of $4f^1$ states calculated at the single-point MS-CASPT2(1e,17o) level along the reaction coordinate shown in Figure S3.

Reaction Coordinate (Å amu$^{1/2}$) [(a)]	Relative energy ΔE (eV) [(a)]								Energy splitting of $4f^1$ states (eV) [(b)]
	D_0	D_1	D_2	D_3	D_4	D_5	D_6	D_7	
3.1638	0.0000	0.0000	0.0656	0.0667	0.1942	0.1942	0.2150	4.1707	0.2150
2.9941	0.0047	0.0047	0.0704	0.0715	0.1982	0.1991	0.2204	4.1768	0.2157
2.5839	0.1179	0.1179	0.1815	0.1827	0.2956	0.3187	0.3519	4.2545	0.2340
2.0564	0.4254	0.4255	0.4784	0.4807	0.5849	0.6425	0.6866	4.4257	0.2612
1.6211	0.9054	0.9060	0.9495	0.9540	1.0627	1.1344	1.1824	4.7700	0.2770
1.4405	1.5511	1.5523	1.5934	1.6002	1.7090	1.7809	1.8304	5.3274	0.2793
1.2484	2.5418	2.5439	2.5811	2.5915	2.6953	2.7699	2.8211	6.2120	0.2793
1.0281	3.6397	3.6433	3.6738	3.6905	3.7820	3.8648	3.9180	7.1517	0.2783
0.8781	4.2769	4.2821	4.3064	4.3289	4.4089	4.5007	4.5546	7.6409	0.2777
0.7692	4.6779	4.6849	4.7038	4.7313	4.8014	4.9016	4.9569	7.9010	0.2790
0.6507	5.0467	5.0570	5.0692	5.1029	5.1614	5.2721	5.3270	8.0702	0.2803
0.4510	5.5344	5.5570	5.5582	5.6025	5.6367	5.7713	5.8268	8.0574	0.2924
0.3763	5.6745	5.7035	5.7097	5.7531	5.7756	5.9227	5.9780	7.9446	0.3035
0.2962	5.8005	5.8455	5.8559	5.8981	5.9067	6.0684	6.1241	7.7611	0.3236
0.2089	5.9071	5.9852	5.9983	6.0389	6.0306	6.2110	6.2667	7.5011	0.3596
0.1117	5.9893	6.1321	6.1467	6.1551	6.1852	6.3590	6.4148	7.1702	0.4255
0.0000	6.0217	6.2864	6.2920	6.3084	6.3441	6.5209	6.5757	6.7749	0.5540

(a) These values are used for describing the PECs of Figure S4 (b and c).
(b) The energy splitting of $4f^1$ states corresponds to the energy difference between D_0 and D_6 states.

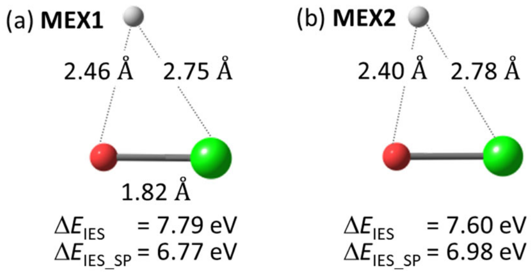

Figure S4. Comparison of the MEXs between the lowest $4f^0X$ state and the lowest $4f^1$ state (MEX1; a), and between the lowest $4f^0X$ state and the highest $4f^1$ state (MEX2; b). MEX1 is identical to the IES-optimized MEX shown in Figure 3(e). MEX2 is located between the lowest $4f^0X$ state described at the IES level and the highest $4f^1$ state described at the ES level, with an energy shift of 0.2150 eV, corresponding to the energy splitting among the $4f^1$ states at the GM geometry. ΔE_{IES} and ΔE_{IES_SP} represent the relative energies (in eV) obtained using the IES method and single-point MS-CASPT2 calculations at the IES-optimized geometry, respectively. The reference for the relative energies is the energy of the GM. Bond distances are in Å. Comparison of MEX1 and MEX2 indicates that, irrespective of which state of $4f^1$ is considered, the MEX with the $4f^0X$ state exhibits similar geometries and energetics.

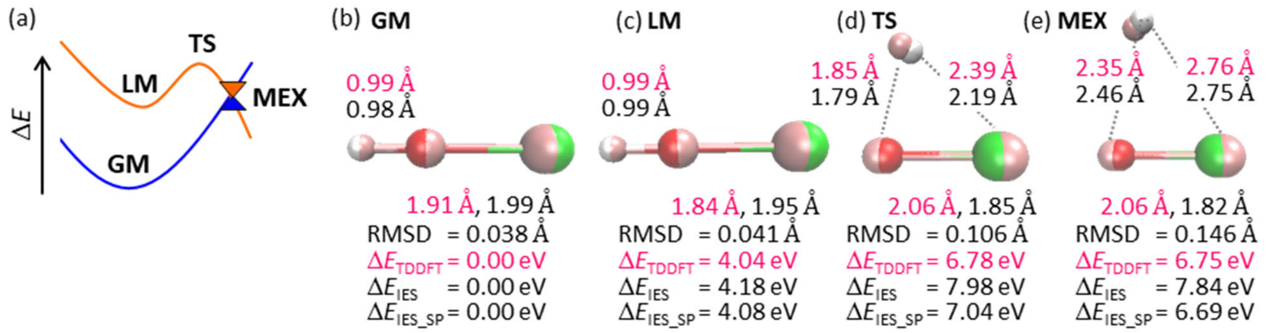

Figure S5. Comparison of the optimized geometries of $CeOH^{2+}$ calculated with the IES method (in atomic-color-code) and TDDFT (in pink; ωB97XD/ECP28MWB, cc-pVDZ). The atomic distances (in Å) obtained with the TDDFT and IES methods are in pink and black, respectively. The RMSDs between the TDDFT- and IES-optimized geometries are in Å. ΔE_{TD}, ΔE_{IES}, ΔE_{IES_SP} represent the relative energies (in eV) obtained with the TDDFT, IES, and single-point TDDFT at the IES-optimized geometries. The IES parameter was determined to reproduce the excitation energy at the GM with the TDDFT. To optimize the MEX with the TDDFT, the PES of the excited state was -5.0×10^{-4} Hartree shifted to avoid discontinuities on TDDFT-PES around MEX. The IES parameter used was 6.374622 Hartree.

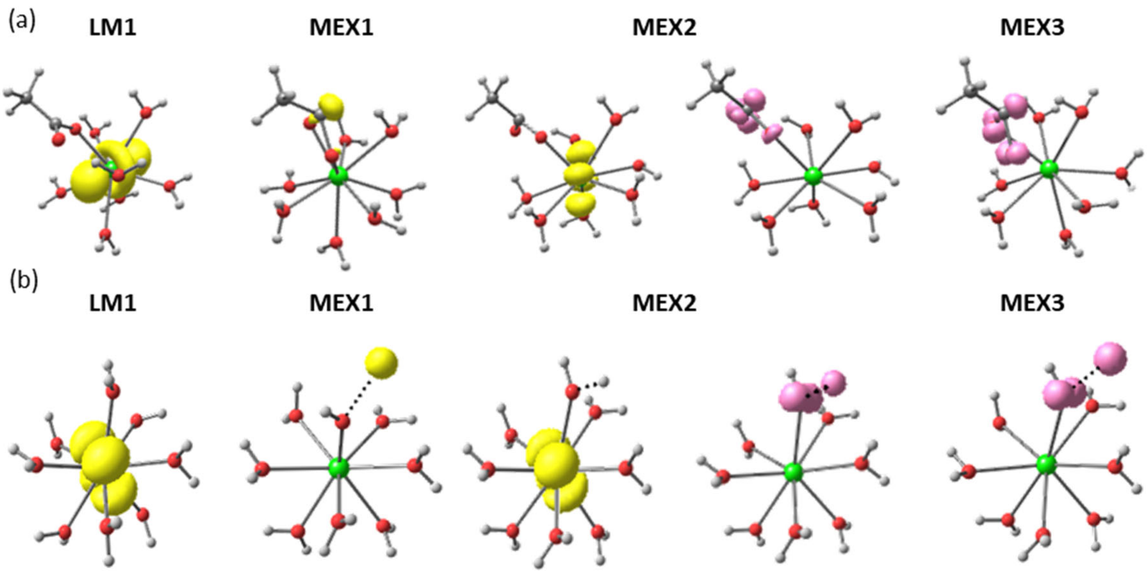

Figure S6. Spin densities of the LMs and MEXs for the carboxylate complex (a) and the hydrate complex (b) calculated at the ωB97XD/ECP46MWB for $4f^0X$ excited states (in yellow) and ωB97XD/ECP47MWB for ligand-localized T1 states (in pink).

2. Cartesian coordinates and energies of critical structures

The Cartesian coordinates (in Å) and the electronic energies (in Hartree) of the optimized structures are shown. The electronic energy used for geometry optimization is E_{opt}. The single-point energy E_{SP} is also shown.

2.1 $CeOH^{2+}$ optimized with the IES method

The IES parameter was 6.372629 Hartree. The single-point energies $E_{SP(PT2)}$ and $E_{SP(PT2:\ 7th)}$ represent the electronic energies of the ground and the doublet 7th excited state calculated at MSCASPT2, respectively.

GM: E_{opt} = -107.431416026859 / $E_{SP(PT2)}$ = -548.996073382338

Ce	-0.040130624572	-0.569553478548	0.194651801415
H	-2.752048266436	-0.963067906037	-0.954604193735
O	-1.858886981872	-0.832976552832	-0.575958336055

LM: E_{opt} = -113.652256898585 / $E_{SP(PT2:7th)}$ = -548.850014563363

Ce	-0.075357662676	-0.556416035520	0.185030910480
H	-2.749605483065	-0.949309647057	-0.949670592107
O	-1.849761962303	-0.819671520533	-0.568606762554

TS: E_{opt} = -113.512709020241 / $E_{SP(PT2:7th)}$ = -548.722565731628

Ce	-0.193848581193	-0.438573384832	0.174091223441
H	-2.005026484082	0.739374770215	-0.174414444341
O	-1.785444322936	-0.987794418010	-0.592962004639

MEX: E_{opt} = -107.145234702478 / $E_{SP(PT2:7th)}$ = -548.747100368828

Ce	-0.066345819573	-0.567046540343	0.185377814351
H	-2.013669503543	1.366772903753	0.004761923341
O	-1.675219372884	-1.003835448409	-0.555568132691

2.2 $CeOH^{2+}$ optimized with the MSCASPT2 method

GM: E_{opt} = -548.99974694264

Ce	-0.084767807893	-0.575992292912	0.175746600087
H	-2.733918322397	-0.960376385968	-0.946903416444
O	-1.832379742589	-0.829229258536	-0.564753912018

LM: E_{opt} = -548.85726019614

Ce	-0.125802363786	-0.581914783853	0.158366247154
H	-2.718691895147	-0.958122060289	-0.940437708964
O	-1.806571613948	-0.825561093276	-0.553839266566

TS: E_{opt} = -548.74441242527

Ce	-0.236980880340	-0.477619878649	0.146276536396
H	-2.011119615416	0.627620967582	-0.209242251633
O	-1.745155863737	-0.894643969424	-0.550496705633

MEX: E_{opt} = -548.748240558316

Ce	-0.074724623744	-0.643185944081	0.171019467914
H	-2.258014534466	1.364177245735	-0.090186382598
O	-1.641857679161	-1.242160822708	-0.622179805582

2.3. The carboxylate complex

The IES parameter was 6.376296 Hartree. The single-point energies $E_{SP(DFT:doublet)}$, $E_{SP(DFT:quartet)}$, and $E_{SP(TDDFT:doublet\ n th)}$ represent the electronic energies of the lowest doublet state, lowest quartet state, and the doublet nth excited state, respectively, as calculated using the DFT/TDDFT method.

LM0 E_{opt} = -795.634568773481 / $E_{SP(DFT:doublet)}$ = -1238.48285463

Ce	-0.077654561997	-0.035566458365	0.187970417538
O	0.508769641721	-1.979479012377	-1.309871699294
O	2.087501579137	-0.493412287423	-0.969051751443
C	1.686441655331	-1.565114257229	-1.527192222745
C	2.593702176607	-2.305252080887	-2.466791932555
H	2.265427878317	-3.343863398132	-2.587778067073
H	2.549385698443	-1.805816653608	-3.447052490558
H	3.630940539802	-2.261994508732	-2.112465731558
O	-0.010914282110	1.030863063691	-2.147889320268
H	0.820791963538	1.060039616670	-2.640233219528
H	-0.366975570037	1.930568679047	-2.176968245407
O	1.557977410476	1.894421794591	0.412072870499
H	2.429145458459	1.805887223834	0.000799152625
H	1.699516720326	2.374219618104	1.239144788302
O	-2.187308687081	-0.406975903413	-1.193877872258
H	-2.730175573486	-1.197587128308	-1.070999930152
H	-2.086939502370	-0.305595452955	-2.151252765736
O	-1.373823344037	2.175679365404	-0.027780789507
H	-1.075013754069	3.030250644705	0.311060753577
H	-2.339617246150	2.221828880548	-0.033010787476
O	-0.135438701056	1.023595522306	2.537688354906
H	0.478240339389	0.735322360814	3.227125138399
H	-0.918147135670	1.342568956010	3.006449397136
O	-1.065354662124	-2.315077828784	0.823931485018
H	-1.217259587730	-2.727960997908	1.683425852018
H	-0.636985576272	-2.984233302307	0.267638973288
O	1.702835726324	-1.040857319225	1.732678275467
H	1.628293418796	-1.729201471997	2.406559631291
H	2.549336810546	-1.194425806048	1.287826401505

LM1 E_{opt} = -801.855127074788 / $E_{SP(TDDFT:doublet\ 7th)}$ = -1238.30500446

Ce	0.027950844741	0.241849123632	0.214005708221
O	0.845822877604	-1.387876058274	-1.211072992759
O	2.995276665363	-1.724504625602	-0.699993350698
C	2.019764657148	-1.874928347440	-1.455458192266
C	2.149501405920	-2.659255372101	-2.734365113021
H	1.399852590917	-3.462992368201	-2.747125482685
H	1.933067391717	-1.996648213424	-3.585112194386
H	3.156099845266	-3.079509471518	-2.835789300707
O	0.285446613923	1.602361649583	-1.858077583251
H	1.151876409539	1.842194782152	-2.214666080242
H	-0.256253303398	1.352836614022	-2.621098857628
O	1.276362359745	2.263430903352	0.842910412801
H	2.217502105192	2.242106091296	1.063324518825
H	0.910948891606	3.054264709438	1.263390851217
O	-1.622996463418	-0.457154968614	-1.516063688736

H	-2.557538772742	-0.661701155741	-1.377996201195
H	-1.239703145390	-1.208649496600	-1.997590544801
O	-1.544977824286	2.153842348453	0.169851849920
H	-1.510496219579	2.803563206396	-0.546777277504
H	-2.441390597140	2.190363657049	0.528614929666
O	-0.432581630210	0.591063083918	2.599683637649
H	0.176915306897	0.925593254493	3.272395311383
H	-1.265506867782	0.412785062206	3.056118442586
O	-0.611648758971	-1.997186524716	1.070996019392
H	-1.486492708103	-2.229710263019	1.409872922314
H	-0.332148557408	-2.740022174974	0.515581107764
O	2.028985308554	-0.512506023743	1.340711765739
H	1.985037099779	-1.100393882911	2.107669547352
H	2.545552373472	-1.011983077113	0.618428194077

MEX1 E_{opt} = -795.406500598445 / $E_{SP(TDDFT:doublet\ 7th)}$ = -1238.25136839, $E_{SP(TDDFT:doublet\ 6th)}$ = -1238.26582485

Ce	0.166747907807	-0.109336858128	-0.011452930157
O	0.626083874115	-1.949598932357	-0.935021234372
O	2.070551422978	-0.222930252129	-0.914514859090
C	2.054544610662	-1.678186411079	-1.090037526374
C	2.548185775312	-2.056445964301	-2.447283827745
H	2.496000709156	-3.147311249877	-2.585021512538
H	1.949645677379	-1.575475962113	-3.241415500034
H	3.597581940904	-1.745314221049	-2.566747495767
O	0.092663683077	1.004206708316	-2.184563501466
H	0.975183297064	0.968976317232	-2.587074672616
H	-0.312408846477	1.851625168327	-2.417802920889
O	1.532302518800	1.843013257428	0.456041485284
H	2.429180626636	1.647069909407	0.133162235053
H	1.612958800454	2.321695927841	1.293521758712
O	-1.974103347728	-0.447550558460	-1.139949104166
H	-2.530867235778	-1.188571272867	-0.855459912725
H	-2.019024414011	-0.417626437577	-2.108086124076
O	-1.292998182608	1.946526665647	0.072931386230
H	-1.024284801416	2.870779648213	0.168725990765
H	-2.227458428731	1.946678285319	-0.179328332280
O	-0.294679015436	0.553217352702	2.309612112213
H	0.237634499819	0.406243202702	3.104173837115
H	-0.951966169843	1.231492216255	2.525045026792
O	-1.115586635351	-1.886292171180	1.039474208057
H	-1.472718954387	-1.942130005882	1.937584233127
H	-0.825050267100	-2.770669493072	0.762735502158
O	1.763512877140	-1.129120283153	1.571240773860
H	1.518911712041	-1.788905548473	2.235714660618
H	2.336155198537	-1.603227179674	0.915950910301

MEX2 E_{opt} = -795.453168342318 / $E_{SP(TDDFT:doublet\ 7th)}$ = -1238.30278388, $E_{SP(DFT:quartet)}$ = -1238.30936047

Ce	-0.314895734332	0.006211757541	0.090599547237
O	1.203288378474	-1.129399284703	-1.319872124328
O	3.163664896113	-0.590870025112	-0.440601007287
C	2.514472355767	-1.159590883881	-1.434124536237
C	3.199641777824	-2.116518444244	-2.344233590683

H	2.674703925161	-2.156496222621	-3.309563598288
H	4.243839229101	-1.817972817109	-2.511226546985
H	3.200826381537	-3.138833114405	-1.915592813789
O	-0.739760914891	0.750317528224	-2.343697866033
H	-0.064201020806	0.427133937828	-2.957632950203
H	-0.811602253666	1.701367216335	-2.509086615951
O	1.643796426761	1.601084047331	0.010499976572
H	2.456171729843	1.083538368868	-0.184327021566
H	1.866273679775	2.191623561218	0.743190899704
O	-2.800805639416	0.010697053270	-0.578555510480
H	-3.521416511025	-0.388260164426	-0.072221333321
H	-2.998177777780	-0.168966058994	-1.509351060999
O	-1.347023113467	2.352730576145	-0.043939404121
H	-1.050930727772	3.152661663123	0.410261834717
H	-2.312612555206	2.405622466471	-0.081866599014
O	-0.190151466449	1.035957632646	2.460658309431
H	0.431356725029	0.542410531532	3.016627754001
H	-0.943309055751	1.248725636991	3.027654986752
O	-0.730242609027	-2.473363184530	0.617526826799
H	-1.444820474729	-2.782963024271	1.190866442364
H	-0.642757142499	-3.142335040541	-0.076250238920
O	1.490629852704	-0.974514116451	1.678558263538
H	1.296180153374	-1.901138371571	1.883838495965
H	2.332089384284	-0.997628762667	1.174227840075

MEX3 E_{opt} = -795.466354459480 / $E_{SP(DFT:doublet)}$ = -1238.31291912 (doublet), $E_{SP(DFT:quartet)}$ = -1238.31690372

Ce	-0.219480200782	0.000399869030	0.184221435920
O	0.660116653162	-1.875013598862	-1.376252393866
O	1.821645722967	-0.349529104141	-1.378849655868
C	2.025591246569	-1.708320196772	-1.242714739106
C	2.924937670887	-2.395719037534	-2.222755261988
H	2.887617389719	-3.483126821694	-2.066728002105
H	2.616606170584	-2.169568289775	-3.258646268993
H	3.962320065905	-2.063029003119	-2.076215559305
O	-0.278665491831	1.125191940338	-2.165388913440
H	0.548954199857	0.960749754247	-2.642794196275
H	-0.484410488905	2.061938658321	-2.284094029531
O	1.424064336484	1.993372579344	0.398261436601
H	2.268863301299	2.016001727710	-0.071169225166
H	1.627439076300	2.216898867306	1.318282769930
O	-2.299521009222	-0.454887226582	-1.259744308257
H	-2.828508293602	-1.263301706234	-1.249535419517
H	-2.200359758755	-0.201877300393	-2.188940730698
O	-1.377070614649	2.302309816748	0.212756430874
H	-0.877883015739	3.125614317209	0.298726690085
H	-2.307693403913	2.535379494190	0.324167375616
O	0.042746684985	1.000456087913	2.609806205717
H	0.531147697520	0.559343177075	3.317786829531
H	-0.684885944400	1.458739408711	3.052307990565
O	-1.184650289136	-2.330836451420	0.789619685426
H	-1.488013816093	-2.616529701758	1.661554628254
H	-0.829173503161	-3.121997186638	0.362095398997

O	1.775211287049	-1.086408280757	1.413018306716
H	1.689337169320	-1.726669042435	2.132570430270
H	2.220415990597	-1.576750892008	0.670807755619

2.5. The hydrate complex

The IES parameter was 6.375596 Hartree. The single-point energies $E_{SP(DFT:doublet)}$, $E_{SP(DFT:quartet)}$, and $E_{SP(TDDFT:doublet\ n th)}$ represent the electronic energies of the lowest doublet state, lowest quartet state, and the doublet *n*th excited state, respectively, as calculated using the DFT/TDDFT method.

LM0 E_{opt} = -643.439772024136 / $E_{SP(DFT:doublet)}$ = -1086.29126686

Ce	0.092081161118	-0.038640068752	0.114783238722
O	0.052273186643	1.456766007990	-2.019072933584
H	0.544375653299	1.229094626981	-2.819850230085
H	-0.642498850665	2.064322726734	-2.307024858775
O	0.852064862239	2.415061660977	0.512475606829
H	1.160315589212	2.890999864585	-0.272281785143
H	1.428472376375	2.704250047308	1.233146947042
O	-1.972003462281	-0.660697395028	-1.297306961717
H	-2.785340123044	-1.017998055607	-0.915560750141
H	-2.202934302248	-0.353101757026	-2.183706292973
O	-1.798555038893	1.639559981712	0.505412297229
H	-1.582804676499	2.578003568724	0.600590193394
H	-2.715507595560	1.597259946993	0.204478824070
O	-0.215124037103	0.528369549245	2.612791403994
H	0.439437590632	0.277024633473	3.279039861337
H	-0.680011371837	1.298225692224	2.966929766247
O	-1.667938621036	-1.453197091373	1.336473194412
H	-1.788291020291	-1.238181159925	2.272830564716
H	-1.807781513904	-2.406857390525	1.259820096350
O	1.404345304543	-1.636942376066	1.667338081608
H	1.141886036244	-2.529924321382	1.927608508790
H	2.362844778917	-1.603539199900	1.790344043097
O	0.169596636529	-2.409912427962	-0.887309960815
H	-0.420028368112	-2.625838833744	-1.622992211631
H	0.941408892637	-2.982957776256	-0.985283231176

LM1 E_{opt} = -649.652608845887 / $E_{SP(TDDFT:doublet\ 7th)}$ = -1086.13363975

Ce	-0.245758561237	-0.009939438118	0.157623318218
O	0.504273243608	0.708263018936	-2.129599991240
H	1.409402773093	0.566748327357	-2.441744892696
H	0.210839060152	1.533934664361	-2.541368868419
O	1.121824390922	2.036689690824	0.598246159960
H	2.017223897397	2.180181214918	0.260357205228
H	1.118065501708	2.381866708441	1.503433048285
O	-2.019312562141	-0.583616767306	-1.479111147086
H	-2.928245055944	-0.805987719243	-1.232108181579
H	-2.058323499013	-0.233247927285	-2.380631098334
O	-1.689780803447	1.990684064803	0.526174788409
H	-1.519696580960	2.846032652880	0.106299741298
H	-2.642482765943	1.953515573290	0.689025451667
O	-0.257987022731	0.550478122944	2.577728514566
H	0.311302636906	0.100613245674	3.218295059429

H	-1.045497261105	0.834723167828	3.063105701822
O	-1.530546525629	-1.896012009619	1.112107597259
H	-2.337096615681	-1.831788957236	1.641535141674
H	-1.182087688231	-2.788966288027	1.246575290729
O	1.773201176004	-0.949930757855	1.267343255249
H	1.805057434980	-1.790053808389	1.747105450769
H	2.669727244524	-0.804346163721	0.932858911862
O	0.317157827534	-2.138468641013	-1.035782314150
H	-0.320754754997	-2.475159372404	-1.682248163937
H	1.192411302755	-2.340848760114	-1.395819119766

MEX1 E_{opt} = -643.205906197870 / $E_{SP(TDDFT:doublet\ 7th)}$ = -1086.05321099, $E_{SP(TDDFT:doublet\ 6th)}$ = -1086.08154839

Ce	-0.044752979907	-0.088405264916	0.196361150664
O	0.037071169366	1.202697973126	-1.964845030531
H	0.735098716758	1.113278391987	-2.630027838662
H	-0.489293932465	1.972936549762	-2.222990837416
O	0.946381124633	2.183329965279	0.492183593139
H	1.451996104271	2.653034643266	-0.186780844957
H	1.317528461131	2.432489508179	1.352063137586
O	-1.798791653251	-0.701880959954	-1.452858265285
H	-2.635404613305	-1.070981681133	-1.132794973758
H	-1.970759095604	-0.260330636019	-2.296512819281
O	-1.708988897887	1.679077619845	0.431552388485
H	-1.485895838815	2.618856290317	0.503002220084
H	-2.667710810492	1.595262086405	0.534632953528
O	0.140019057624	0.741945611952	2.556501008550
H	0.538832629677	0.143432267794	3.205980752582
H	-0.600481377208	1.174482730559	3.007654484283
O	-1.543038446004	-1.202078525735	1.172703578281
H	-2.060503895847	-1.173962822116	1.987653288317
H	-2.122512288069	-3.137294870689	0.771979848671
O	1.463721727604	-1.486909371609	1.560801479376
H	1.212820193748	-2.351641544891	1.918939215258
H	2.431970825740	-1.464179401205	1.538660465783
O	0.410553661446	-2.153042079154	-1.049232822382
H	-0.189870422799	-2.449037615658	-1.749175303409
H	1.222804260368	-2.674702364234	-1.116483209436

MEX2 E_{opt} = -643.190025633490 / $E_{SP(TDDFT:doublet\ 7th)}$ = -1086.04063805, $E_{SP(DFT:quartet)}$ = -1086.04208700

Ce	-0.169905492673	-0.002447999108	0.124866283414
O	0.444075076616	0.798096327898	-2.212784368200
H	1.286168958841	0.622254894677	-2.655117738353
H	0.041679286548	1.536472540754	-2.691168728577
O	0.961916678064	2.271373119277	0.525570396257
H	1.541190330867	2.740800620774	-0.090872764196
H	1.196900361008	2.588928536625	1.408879412170
O	-2.054910224907	-0.709298147169	-1.443166373252
H	-2.919021733077	-0.985189556207	-1.104786668166
H	-2.229913323342	-0.294678029100	-2.299818556953
O	-1.758252095205	2.185505782709	0.649014410285
H	-1.792032476524	3.006956523242	0.108227470063
H	-2.322727919993	2.552509478380	1.826202861539

O	-0.357418080564	0.583058577947	2.568461973304
H	0.217902223418	0.201583050493	3.246590419868
H	-1.029550037080	1.105868086415	3.030450072634
O	-1.343979019928	-1.984726374728	1.179726820021
H	-2.058295938261	-1.978366968785	1.830854840668
H	-1.321398541805	-2.874791915528	0.801573583756
O	1.464413639394	-1.373024612807	1.514299131429
H	1.226538480434	-2.209252416194	1.938338505023
H	2.430078040855	-1.352298674757	1.471052443913
O	0.277162313034	-2.166927147983	-1.125443049029
H	-0.278309189009	-2.389891205007	-1.885973334577
H	1.138482364006	-2.576137990647	-1.286009423594

MEX3 E_{opt} = -643.225799548386 / $E_{SP(DFT:doublet)}$ = -1086.08652228, $E_{SP(DFT:quartet)}$ = -1086.07567790

Ce	-0.202058860994	-0.024587040186	0.090336290380
O	0.155625276011	0.862336469224	-2.347591172554
H	1.003536763903	0.755590979667	-2.802214807180
H	-0.178260201622	1.723826636272	-2.635408764678
O	0.959143854740	2.266636053580	0.551313829536
H	1.551858451005	2.764297760617	-0.028539585476
H	1.189660498402	2.530086408204	1.453336149829
O	-1.910271172145	-0.898301963819	-1.642889013888
H	-2.844256912795	-1.075208043623	-1.463002360982
H	-1.886020532683	-0.465918561692	-2.509250794319
O	-1.622726471544	2.255258618639	0.816953054486
H	-1.741944439361	2.915407797520	0.100479682947
H	-2.310864928082	3.483477383122	2.305673528054
O	-0.432943200527	0.540123221816	2.570479905657
H	0.013939733344	0.059205656872	3.279904102412
H	-1.110636318392	1.087113468950	2.989829339589
O	-1.242888740899	-2.105999752393	1.203099981808
H	-2.042488673104	-2.131035957278	1.746246741497
H	-1.227470294656	-2.938637027979	0.709918114717
O	1.426922074771	-1.392974831869	1.534673744135
H	1.165237515709	-2.210096252181	1.980651149848
H	2.379306458729	-1.297018867284	1.664986701751
O	0.375849494629	-2.245851881232	-1.090691989430
H	-0.124434318243	-2.504418674750	-1.877323247825
H	1.246978624523	-2.656935099042	-1.172002960834
